# Gallstone disease and the risk of cardiac mortality in patients with acute coronary syndrome

**DOI:** 10.3389/fcvm.2022.1033959

**Published:** 2022-11-24

**Authors:** Wen Su, Jie-Gao Zhu, Wei-Ping Li, Hui Chen, Hong-Wei Li

**Affiliations:** ^1^Department of Cardiology, Beijing Friendship Hospital, Capital Medical University, Beijing, China; ^2^Department of General Surgery, Beijing Friendship Hospital, Capital Medical University, Beijing, China

**Keywords:** gallstone disease, acute coronary syndrome (ACS), cardiac mortality, all-cause mortality, cholecystectomy

## Abstract

**Background:**

Gallstone disease is a common gastrointestinal disorder, which has previously been reported to be associated with the incidence of cardiovascular disease. We aimed to investigate the association between gallstone disease and long-term outcomes in patients with acute coronary syndrome (ACS).

**Materials and methods:**

A total of consecutive 13,975 ACS patients were included in this analysis. Gallstone disease in our study included both gallstones and cholecystectomy. The primary endpoint was cardiac mortality. The secondary outcome was all-cause mortality. Relative risks were estimated using Cox proportional hazards regression.

**Results:**

During a median follow-up period of 2.96 years, 518 (4.2%) patients without gallstone disease and 62 (3.6%) patients in those with gallstone disease suffered cardiac death. After multivariable adjustment for established risk factors, subjects with gallstone disease had decreased risks of both cardiac mortality and all-cause mortality [hazard rate ratios (HR) = 0.72, 95% CI: 0.55–0.95 and HR = 0.75, 95% CI: 0.62–0.90, respectively]. In patients with performed cholecystectomy, the associations between gallstones and risks for cardiac mortality and all-cause mortality turned out to be non-significant. HRs varied across subgroups depending on the presence of selected established risk factors.

**Conclusion:**

Presence of gallstone disease was associated with a significantly decreased risk of follow-up mortality in patients with ACS.

## Introduction

Gallstone disease is a common gastrointestinal disorder, which affects about 10 to 20% of the adult population in the world (1, 2). Gallstone disease and cardiovascular disease shared several common risk factors, including diabetes, obesity, dyslipidemia, metabolic syndrome, unhealthy diet, and sedentary lifestyle (2–4). Furthermore, gallstone disease has previously been reported to be associated with the incidence of cardiovascular disease (5–8). Since cholesterol is the principal component of atherosclerotic plaques and most gallstones, acute coronary syndrome (ACS) and gallstone disease could be intrinsically linked *via* cholesterol metabolism (9). However, current evidence on the association between gallstone disease and outcomes in the patients with ACS is still limited. Therefore, the aim of our study was to investigate the association between gallstone disease and long-term outcomes based on this real-world cohort of patients with ACS.

## Materials and methods

The current study was an observational cohort study. The study population consisted of patients diagnosed with ACS who were admitted to Beijing Friendship Hospital from January 2013 to January 2020. The definition of ACS included unstable angina pectoris (UAP), ST elevation myocardial infarction (STEMI), and Non-ST elevation myocardial infarction (NSTEMI). Individuals were excluded from the study if data for gallstone status were missing. A total of 13,975 participants were included in the analysis finally. All data was collected from medical records in the Cardiovascular Center Beijing Friendship Hospital Database Bank (CBD Bank). The study protocol was approved by the Institutional Review Board of the Research Institute for Beijing Friendship Hospital, Capital Medical University (IRB number 2019-P2-068-01), and complied with the ethical guidelines of the 1975 Declaration of Helsinki.

### Clinical assessments and follow-up

Clinical characteristics including demographic data, medical history, laboratory results, and echocardiographic data were collected using an electronic medical recording system. Gallstone disease in our study included both gallstones and cholecystectomy. Gallstones were diagnosed by experienced physicians with abdominal ultrasound inspection (IU22; Philips Medical Systems). Blood samples were collected after an overnight fasting and tested for low-density lipoprotein cholesterol (LDL-C), high-density lipoprotein cholesterol (HDL-C), glycosylated hemoglobin (HbA1c), and bilirubin by a certified laboratory at our institution. The peak concentrations of cardiac troponin I (cTnI) and N-terminal pro-brain natriuretic peptide (NTproBNP) were used to estimate myocardial injury.

Treatment of patients were determined by the attending physician, according to prevailing guidelines and individual need. Follow-up data were collected by reviewing outpatient medical records and telephone interviews. The median follow-up was 2.96 years (interquartile range 1.01–4.07). The primary outcome of this study was cardiac mortality, defined as death due to myocardial infarction, left ventricular failure, cardiac perforation or pericardial tamponade, arrhythmia, or any other death in which a cardiac cause could not be excluded. The secondary outcome was all-cause mortality, including cardiac mortality and non-cardiac mortality.

### Statistics analyses

Continuous variables were presented as medians with interquartile ranges and categorical variables as frequencies with percentages. Comparisons between the two study groups were analyzed using the Kruskal–Wallis rank sum test for continuous variables and the Pearson chi-square test for categorical variables. In order to visualize the association between gallstone disease and the risk of follow-up mortality, we calculated hazard rate ratios (HR) using the Cox proportional hazards models. Two adjustments were performed: (1) an age and sex-adjusted model and (2) a multivariable adjusted model taking into account the prespecified potential confounders: age, sex, body mass index (BMI), smoking habits, alcohol intake, classification of ACS, percutaneous coronary intervention (PCI) therapy, previous myocardial infarction, hypertension, diabetes, and hyperlipidemia. We also examined associations between gallstone disease and risk of cardiac mortality among pre-specified baseline subgroups. All data analyses were performed with SPSS 22.0 (IBM Corp., Armonk, NY, USA) and R (version 3.6.3). *P* < 0.05 was considered statistically significant.

## Results

### Baseline characteristics

At baseline, 12.2% of 13,975 patients with ACS reported the presence of gallstone disease (gallstones without cholecystectomy 8.6%; gallstones with cholecystectomy 3.6%). Baseline characteristics according to the presence of gallstone disease were presented in Table 1. Compared with subjects without gallstone disease, those with gallstone disease were older, more likely to be female, had higher prevalence of hypertension, diabetes, and hyperlipidemia, less likely to smoke tobacco and drink alcohol currently, had higher BMI, more prevalent diagnosis of UAP, less likely to receive PCI, had lower levels of TC, HDL-C, LDL-C, higher levels of HbA1C, lower levels of cTnI and NTproBNP, and less likely to have left ventricular ejection fraction (LVEF) <50%.

**TABLE 1:** Baseline characteristics according to the presence of gallstone disease.

	Without gallstone disease (*n* = 12,265)	With gallstone disease (*n* = 1,710)	*P*
Age, yrs	64 (58–73)	69 (61–76)	<0.001
Male, *n* (%)	8,020 (65.4)	950 (55.6)	<0.001
**Medical history, *n* (%)**			
Myocardial infarction	1,180 (9.6)	10 (10.0)	0.619
Hypertension	8,551 (69.7)	1,300 (76.0)	<0.001
Diabetes	4,269 (34.8)	715 (41.8)	<0.001
Hyperlipidemia	3,759 (30.6)	567 (33.2)	0.035
BMI, kg/m^2^	25.4 (23.4–27.8)	25.8 (23.7–28.2)	<0.001
Current smoking, *n* (%)	4,306 (35.1)	451 (26.4)	<0.001
Alcohol drinking, *n* (%)	3,864 (31.5)	412 (24.1)	<0.001
Diagnosis			<0.001
STEMI, *n* (%)	1,928 (15.7)	171 (10.0)	
NSTEMI, *n* (%)	1,989 (16.2)	210 (12.3)	
UAP, *n* (%)	8,348 (68.1)	1,329 (77.7)	
Systolic BP, mmHg	130 (120–141)	130 (120–141)	0.14
Heart rate, bpm	70 (63–79)	70 (63–78)	0.3
Multivessel disease, *n* (%)	6,384 (52.1)	894 (52.3)	0.023
Left main lesion, *n* (%)	1,084 (8.8)	141 (8.2)	0.755
PCI therapy, *n* (%)	6,353 (51.8)	770 (45.0)	<0.001
**Medical treatment**			
Antiplatelet, *n* (%)	11,278 (92.0)	1,566 (91.6)	0.595
Beta-blockers, *n* (%)	8,274 (67.5)	1,156 (67.6)	0.906
ACE inhibitors, *n* (%)	6,605 (53.9)	918 (53.7)	0.896
Statins, *n* (%)	10,487 (85.5)	1,472 (86.1)	0.098
Total bilirubin, μmol/L	11.2 (8.4–15.4)	11.6 (8.5–16.1)	0.015
Direct bilirubin, μmol/L	4.1 (3.0–5.7)	4.4 (3.0–5.9)	<0.001
Indirect bilirubin, μmol/L	7.2 (5.0–10.1)	7.4 (5.2–10.3)	0.055
Amidotransferase, U/L	20 (14–28)	19 (14–27)	0.03
Creatinine, μmol/L	74 (64–88)	73 (64–89)	0.784
TC, mmol/L	4.18 (3.52–4.9)	4.03 (3.45–4.76)	<0.001
HDL-C, mmol/L	1.06 (0.91–1.25)	1.04 (0.89–1.22)	0.01
LDL-C, mmol/L	2.35 (1.87–2.88)	2.25 (1.79–2.78)	<0.001
Triglyceride, mmol/L	1.36 (0.99–1.94)	1.35 (1.01–1.88)	0.773
HbA1c,%	6.0 (5.6–7.0)	6.2 (5.7–7.2)	<0.001
cTnI, ng/mL	2.9 (0.3–13.0)	1.8 (0.1–10.0)	0.006
NTproBNP, pg/Ml	303 (94–1479)	245 (93–1,156)	0.017
LVEF < 50%, *n* (%)	1,312 (10.7)	142 (8.3)	0.003

Values are median (interquartile range) unless otherwise indicated.

BMI, body mass index; BP, blood pressure; cTnI, cardiac troponin I; eGFR, estimated glomerular filtration rate; HbA1C, glycosylated hemoglobin; HDL-C, high density lipoprotein cholesterol; LDL-C, low density lipoprotein cholesterol; LVEF, left ventricular ejection fraction; NSTEMI, Non-ST elevation myocardial infarction; NTproBNP, N-terminal pro-brain natriuretic peptide; PCI, percutaneous coronary intervention; STEMI, ST elevation myocardial infarction; TC, total cholesterol; UAP, unstable angina pectoris.

### Study outcomes

During a median follow-up of 2.96 years, we documented 580 incident cases of cardiac death [518 (4.2%) in patients without gallstone disease and 62 (3.6%) in those with gallstone disease]. The cumulative incidence of all-cause mortality according to the presence of gallstone disease were 980 (8.0%) and 121 (7.1%) for participants without and with gallstone disease, respectively.

**FIGURE 1:**
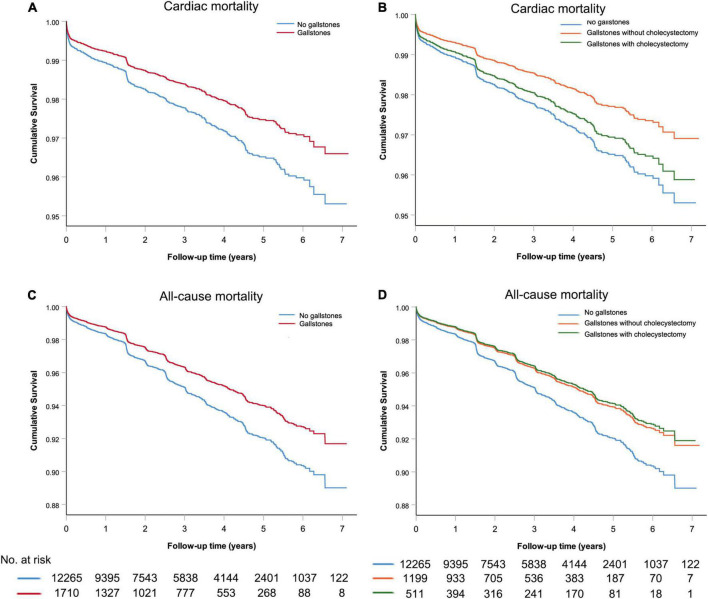
The cumulative survival analysis for acute coronary syndrome patients with or without gallstone **(A,C)** and subgroup analysis for gallstone patients with or without cholecystectomy **(B,D)**.

Table 2 shows the estimated HRs of follow-up mortality regarding the presence of gallstone disease. In age and sex-adjusted analyses, decreased risks of cardiac mortality and all-cause mortality were observed in participants with gallstone disease. After additional adjustment for other potential confounders, including BMI, smoking habits, alcohol intake, classification of ACS, PCI therapy, previous myocardial infarction, hypertension, diabetes, and hyperlipidemia, the associations were only slightly attenuated and remained significant for both cardiac mortality and all-cause mortality (HR = 0.72, 95% CI: 0.55–0.95 and HR = 0.75, 95% CI: 0.62–0.90, respectively). Individuals were additionally divided according to gallstones with and without cholecystectomy. Interestingly, in patients with performed cholecystectomy, the associations between gallstones and risks for cardiac mortality and all-cause mortality turned out to be non-significant (HR = 0.88, 95% CI: 0.57–1.34 and HR = 0.73, 95% CI: 0.52–1.02, respectively). The cumulative survival proportions according to the presence of gallstone disease were shown in Figure 1.

**TABLE 2:** Multivariable adjusted hazard rate ratio (HR) of cardiac mortality and all-cause mortality regarding the presence of gallstone disease (*n* = 13,975).

	*N*	Cases (%)	Model 1^a^ HR (95% CI)	Model 2^b^ HR (95% CI)
**Cardiac mortality**				
No gallstone disease	12,265	518 (4.2)	Reference	Reference
All gallstone disease	1,710	62 (3.6)	0.67 (0.52, 0.88)	0.72 (0.55, 0.95)
Gallstones without cholecystectomy	1,199	40 (3.3)	0.63 (0.45, 0.86)	0.65 (0.47, 0.91)
Gallstones with cholecystectomy	511	22 (4.3)	0.77 (0.50, 1.19)	0.88 (0.57, 1.34)
**All-cause mortality**				
No gallstone disease	12,265	980 (8.0)	Reference	Reference
All gallstone disease	1,710	121 (7.1)	0.70 (0.58, 0.85)	0.75 (0.62, 0.90)
Gallstones without cholecystectomy	1,199	86 (7.2)	0.72 (0.58, 0.90)	0.75 (0.60, 0.94)
Gallstones with cholecystectomy	511	35 (6.8)	0.66 (0.47, 0.93)	0.73 (0.52, 1.02)

CI, confidence interval; HR, hazard rate ratio; ^a^, Adjusted for age and sex; ^b^, additionally adjusted for body mass index, smoking habits, alcohol intake, classification of acute coronary syndrome, percutaneous coronary intervention therapy, previous myocardial infarction, hypertension, diabetes, and hyperlipidemia.

**FIGURE 2:**
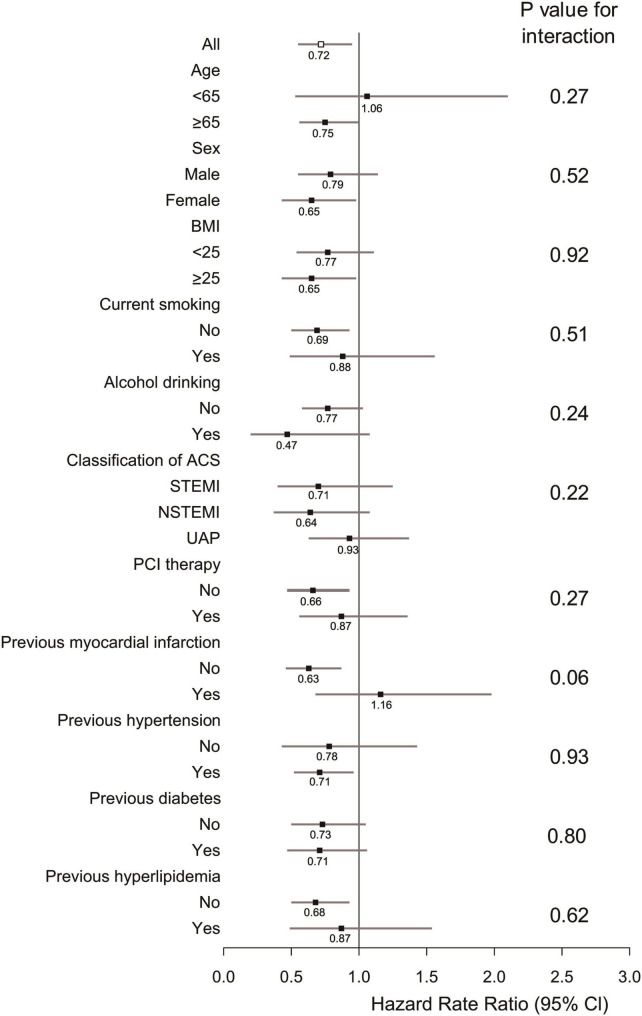
Subgroup analyses of association between gallstone disease and cardiac mortality according to potential baseline risk factors. Adjustments were made for age, sex, body mass index (BMI), smoking habits, alcohol intake, classification of acute coronary syndrome (ACS), percutaneous coronary intervention (PCI) therapy, previous myocardial infarction, hypertension, diabetes, and hyperlipidemia.

We also investigated the associations between gallstone disease and cardiac mortality according to potential baseline risk factors. Although no significant interaction was detected, HRs varied across some subgroups. Notably, the association of gallstone disease with cardiac mortality appeared to be stronger in women, and individuals with higher BMI, no current smoking habit, previous hypertension, and individuals without PCI therapy and previous hyperlipidemia (Figure 2).

## Discussion

The present study revealed that presence of gallstone disease was associated with a significantly decreased risk of follow-up mortality in patients with ACS, and such association was independent of traditional cardiovascular risk factors. To our knowledge, this was the first cohort study examining this association.

Previous studies have assessed the impact of gallstones on incidence of cardiovascular disease. Although results were not consistent (10), most of the studies reported that gallstones were associated with increased risk of incident cardiovascular disease (5–8). Since cholesterol gallstones are formed on the basis of cholesterol supersaturated bile, and account for 90–95% of all gallstones (2), it was reasonable to suspect that presence of ACS might be related to a higher prevalence of gallstones. In our study, the prevalence of gallstone disease among patients with ACS was 12.2%, higher than that among general Asian population (11).

According to current guidelines, symptomatic gallstones require therapy which includes open or laparoscopic cholecystectomy. Asymptomatic gallstones which account for the majority of gallstones should be managed expectantly (2). It was argued that cholecystectomy might stimulate an increased cholesterol excretion through the gastrointestinal tract and therefore might improve cardiovascular risk and clinical outcomes (12, 13). However, we found gallstones without cholecystectomy seemed to be a protector of cardiac mortality in patients with ACS. The main reason for this discrepancy was thought to be the differences between the study populations.

There are potential mechanisms accounting for the association between gallstones and better outcomes in ACS patients. First, gallstones may promote lifestyle modifications in these patients. From the baseline characteristics, we found that patients with gallstone disease were less likely to smoke tobacco and drink alcohol currently, had lower levels of cholesterol on admission despite of the higher prevalence of hypertension, diabetes, and hyperlipidemia which were diagnosed based on the records of medical history. Although we possessed no specific information on the diet and physical activity, it seemed reasonable to suspect that abdominal discomfort caused by unhealthy lifestyle in gallstone carriers might promote lifestyle modifications in ACS patients. Secondly, Statins can decrease hepatic cholesterol synthesis and consequently biliary cholesterol secretion, and may thereby lead to less symptoms in patients with gallstones (14–16). It is possible to speculate that gallstone carriers might have better compliance of long-term statin therapy, which can exert beneficial effects on outcomes among ACS patients (17). Thirdly, from a basic pathophysiological perspective, in gallstone patients, Abcg5/g8 may be elevated expression by the role of gut microbiota or genetic variation (18–20). As mentioned above, abnormally elevated expression of Abcg5/g8 can reduce cholesterol concentration in blood by transporting more cholesterol to the intestine and bile (21). At the same time, Niemann–Pick C1-Like Protein1, a transmembrane protein highly expressed in the intestinal tract in gallstone patients, could reduce the intestinal cholesterol uptake (22). Therefore, patients with gallstone may have relatively lower plasma LDL cholesterol level which is the risk factor for atherosclerosis and ischemic vascular disease. There were also some studies found that the serum deoxycholic acid was higher in patients with gallstone (23). Deoxycholic acid-G protein-coupled bile acid receptor (one of the receptors sensing bile acids to mediate their biological functions) signaling pathway activation decreases inflammation to provide beneficial effects in patients with myocardial infarction (24). However, further studies are needed to clarify whether there are direct effects of gallstones on outcomes of ACS patients.

Several limitations in our study should be taken into account: First, the analyses were not adjusted for potentially relevant lifestyle parameters such as high-fat or high-carbohydrate diet, physical activity, or coffee consumption. Secondly, our study could not propose cause-and-effect relationships between gallstones and decreased mortality. Thirdly, we had only information about the presence of gallstone, but no specific information on the subtypes of gallstone.

## Conclusion

In summary, the presence of gallstone disease was associated with a significantly decreased risk of mortality in patients with ACS. Additional studies are needed to confirm the association and to elucidate the potential protective mechanisms.

## Data availability statement

The original contributions presented in this study are included in the article/supplementary material, further inquiries can be directed to the corresponding author.

## Ethics statement

The studies involving human participants were reviewed and approved by the Institutional Review Board of the Research Institute for Beijing Friendship Hospital, Capital Medical University. The patients/participants provided their written informed consent to participate in this study.

## Author contributions

WS designed the study and wrote the manuscript. J-GZ contributed to the conception, analysis, and interpretation of the data. W-PL reviewed and revised the manuscript. HC and H-WL participated in collection of the data. All authors read and approved the final work.
